# Genome-wide identification and transcriptional analysis of folate metabolism-related genes in maize kernels

**DOI:** 10.1186/s12870-015-0578-2

**Published:** 2015-08-19

**Authors:** Tong Lian, Wenzhu Guo, Maoran Chen, Jinglai Li, Qiuju Liang, Fang Liu, Hongyan Meng, Bosi Xu, Jinfeng Chen, Chunyi Zhang, Ling Jiang

**Affiliations:** Biotechnology Research Institute, Chinese Academy of Agricultural Sciences, Beijing, People’s Republic of China; Huazhong Agricultural University, Wuhan, People’s Republic of China; Beijing Institute of Pharmacology and Toxicology, Beijing, People’s Republic of China; National Key Facility for Crop Gene Resources and Genetic Improvement (NFCRI), Beijing, People’s Republic of China; Southwest University of Science and Technology, Mianyang, People’s Republic of China

**Keywords:** Maize, Folate metabolism, C1 metabolism, Expression pattern, Folate profiling

## Abstract

**Background:**

Maize is a major staple food crop globally and contains various concentrations of vitamins. Folates are essential water-soluble B-vitamins that play an important role as one-carbon (C1) donors and acceptors in organisms. To gain an understanding of folate metabolism in maize, we performed an intensive *in silico* analysis to screen for genes involved in folate metabolism using publicly available databases, followed by examination of the transcript expression patterns and profiling of the folate derivatives in the kernels of two maize inbred lines.

**Results:**

A total of 36 candidate genes corresponding to 16 folate metabolism-related enzymes were identified. The maize genome contains all the enzymes required for folate and C1 metabolism, characterized by highly conserved functional domains across all the other species investigated. Phylogenetic analysis revealed that these enzymes in maize are conserved throughout evolution and have a high level of similarity with those in sorghum and millet. The LC-MS analyses of two maize inbred lines demonstrated that 5-methyltetrahydrofolate was the major form of folate derivative in young seeds, while 5-formyltetrahydrofolate in mature seeds. Most of the genes involved in folate and C1 metabolism exhibited similar transcriptional expression patterns between these two maize lines, with the highest transcript abundance detected on day after pollination (DAP) 6 and the decreased transcript abundance on DAP 12 and 18. Compared with the seeds on DAP 30, 5-methyltetrahydrofolate was decreased and 5-formyltetrahydrofolate was increased sharply in the mature dry seeds.

**Conclusions:**

The enzymes involved in folate and C1 metabolism are conserved between maize and other plant species. Folate and C1 metabolism is active in young developing maize seeds at transcriptional levels.

## Background

**Fig. 1:**
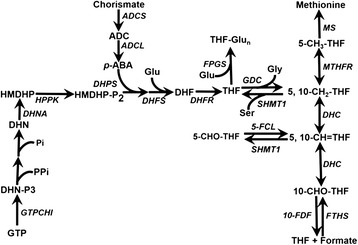
Schematic representation of the key folate and C1 metabolic reactions in maize. Enzymes involved in folate biosynthesis include: aminodeoxychorismate (ADC) synthase (ADCS) and ADC lyase (ADCL) in the chloroplast, GTP cyclohydrolase I (GTPCHI) and dihydroneopterin (DHN) aldolase (DHNA) in the cytosol, hydroxymethyldihydropterin pyrophosphokinase and dihydropteroate synthase (HPPK-DHPS), dihydrofolate synthetase (DHFS), dihydrofolate reductase (DHFR), and folylpolyglutamate synthetase (FPGS) in the mitochondria. Enzymes involved in C1 metabolic pathways include: glycine decarboxylase complex (H protein, GDCH; P protein, GDCP; T protein, GDCT; L protein), serine hydroxymethyl transferase 1 (SHMT1), 5,10-methylenetetrahydrofolate dehydrogenase/5,10-methenyl-tetrahydrofolate cyclohydrolase (DHC), 5, 10-methylenetetrahydrofolate reductase (MTHFR), methionine synthase (MS), 10-formyl THF deformylase (10-FDF), 10-formyltetrahydrofolate synthetase (FTHS), and 5-formyltetrahydrofolate cycloligase (5-FCL) (modified according to the figures from Li et al., [45]; Blancquaert et al., [1]; Hanson and Gregory, [2])

Folates are essential water-soluble B-vitamins, including tetrahydrofolate (THF) and its derivatives. Folates play an important role as one-carbon (C1) donors and acceptors in all types of species. Folate molecules consist of a pteridine ring, a para-aminobenzoate (p-ABA) ring, and a tail of one or more L-glutamate. The C1 substituents attach to the N^5^ position of the pteridine and/or to the N^10^ position of p-ABA to form all types of folate derivatives that have different properties and functions [1, 2]. *De novo* biosynthesis of folate is restricted to plants and microorganisms, but not animals. The reactions required to synthesise tetrahydrofolate are basically the same in plants as in bacteria and fungi [3]. In cytosol, GTP cyclohydrolase I (EC:3.5.4.16, GTPCHI) catalyses the first step during conversion of GTP to dihydroneopterin, and dihydroneopterin (DHN) aldolase (EC:4.1.2.25, DHNA) cleaves the lateral side chain of DHN to form 6-hydroxymethyldihydropterin. In plastids, 4-aminodeoxychorismate (ADC) is produced from chorismate by ADC synthase (EC:2.6.1.85, ADCS) and is esterified to form p-ABA by ADC lyase (EC:4.1.3.38, ADCL). Pterins and p-ABA are subsequently condensed, glutamylated, and reduced to form THF monoglutamate in the mitochondria. In mitochondria, dihydrofolate is converted by hydroxymethyldihydropterin pyrophosphokinase (EC:2.7.6.3, HPPK) and dihydropteroate synthase (EC:2.5.1.15, DHPS), which is a bifunctional enzyme in plants, and then attached to the first glutamate through the action of dihydrofolate synthetase (EC:6.3.2.17, DHFS). Later, dihydrofolate is reduced to THF by dihydrofolate reductase (EC:1.5.1.3, DHFR). THF monoglutamate can be transported to cytosol and plastids, respectively, and become polyglutamylated through the action of folylpolyglutamate synthetase (EC:6.3.2.17, FPGS) in different cellular compartments. During C1 metabolism, polyglutamylated THF is used as a cofactor in glycine (Gly) and 5,10-methylene THF biosynthesis from serine by serine (Ser) hydroxymethyltransferase (EC:2.1.2.1, SHMT), and Ser serves as an alternate donor of C1. THF is recycled back by glycine decarboxylase (EC:1.4.4.2, GDC), which is involved in 5,10-methylene-THF formation from Gly and THF, and the glycine decarboxylase complex consists of four different component proteins; namely, P-(GDCP), H-(GDCH), T-(GDCT), and L-proteins [4]. Then, 5,10-methylene-THF can be reversibly oxidised to 10-formyl THF by the bifunctional 5,10-methylene-THF dehydrogenase/5,10-methenyl-THF cyclohydrolase (EC:1.5.1.5 3.5.4.9, DHC). Compound 10-formyl THF deformylase (EC 3.5.1.10, 10-FDF) can hydrolyse 10-formyl THF to release THF and formate, while 10-formyltetrahydrofolate synthetase (EC:6.3.4.3, FTHS) can consume THF and formate to re-form 10-formyl THF. Besides, 5,10-methylene-THF can be reduced to 5-methyl-THF (5-M-THF) by methylenetetrahydrofolate reductase (EC:1.5.1.20, MTHFR), and 5-methyl-THF can serve as a methyl donor for methionine synthesis (EC:2.1.1.14, MS) from homocysteine. Additionally, 5-formyl THF cycloligase (EC:6.3.3.2, 5-FCL) and 5-formyl THF cycloligase-like protein (5-FCLL) can catalyse 5-formyl THF (5-F-THF) conversion to 5,10-methenyltetrahydrofolate; while SHMT1 promotes the formation of 5-F-THF [5, 6]. Overall, 16 enzymes are involved in folate and C1 metabolism in plants (Fig. 1) [2, 3].

Due to the lack of functional DHNA, HPPK/DHPS, ADCS, ADCL, and DHFS, humans cannot synthesize folate *de novo*, and thus folate fortification in foods such as wheat flour is required [2]. Besides, overexpressing folate biosynthetic and metabolic enzymes originating from plant or non-plant organisms is known to be an effective alternative to enhance folate contents in food crops including tomato, rice, and maize [7–10]. Maize is a major staple food crop globally. To date, few studies on folate metabolism genes in maize are available [11, 12]. For example, the first DHFR-TS gene from maize was cloned and the RNA transcripts for ZmDHFR-TS were shown to accumulate to high levels in developing maize kernels and meristematic tissues [11]. Another gene involved in folate metabolism was characterised in the *brown midrib 2* (*bm 2*) mutant, in which a functional MTHFR gene showed reduced transcript levels. As a result, the mutant showed a reddish-brown colour associated with reductions in lignin concentration and alterations in lignin composition [12]. However, no systematic characterisation of folate metabolism genes in maize has been reported, and how folates flow during maize kernel formation remains unknown. Therefore, identification of folate-related genes at the whole genome level and characterisation of folate metabolism during maize kernel formation could provide a foundation for understanding of the folate metabolism in maize and molecular breeding of folate-fortified maize varieties.

In this study, an intensive *in silico* analysis was performed to screen for genes involved in folate metabolism using all publicly available databases. We found that the maize genome contains all enzymes required for folate and C1 metabolism, which are characterised by highly conserved domains, similar to other species. To further advance our understanding of the folate metabolism in maize, two representative maize inbred lines with significant differences in total folates in mature seeds were chosen to investigate the expression of folate-related genes and the profiling of folate derivatives during kernel formation.

## Results and discussion

### Identification and phylogenetic analysis of putative folate metabolic genes in maize

To understand the folate metabolism in maize, we first investigated the conservation of all folate-related genes between Arabidopsis and maize on a whole-genome scale as the folate metabolism pathway has been well characterised in Arabidopsis compared to other plant species. Folate metabolism involves folate synthesis and the C1 cycle. Enzymes involved in folate synthesis in maize were identified via BLAST using homologs from Arabidopsis. Consequently, eight enzymes were identified (Table 1). One ortholog was identified for HPPK/DHPS and ADCS, respectively, two for GTPCHI, DHNA, DHFS, and FPGS, respectively, three for ADCL, and four for DHFR. Within each group of maize orthologs such as GTPCHI, DHNA, DHFS, and DHFR, the protein similarities were all higher than 90 %. The protein similarity between the two FPGS orthologs was 77.8 %. A rather low protein similarity was observed in between ADCL orthologs (45.3 % for between ADCL1 and ADCL2). These results indicated that the majority of orthologs involved in folate synthesis were conserved in maize.

**Table 1:** Genes involved in folate synthesis identified in maize

Gene identifier	Accession number	Gene function	Enzyme abbreviation	Sequence similarity among orthologs
GRMZM2G062420	A0A096QVI4	GTPCHI	GCH1-1	GCH1-1 and GCH1-2: 92.4 %
GRMZM2G106376	B4FH02	GTPCHI	GCH1-2	
GRMZM2G015588	A0A096PZQ4	DHNA	FOLB1	FOLB1 and FOLB2: 96.3 %
GRMZM2G095579	B4FPQ2	DHNA	FOLB2	
GRMZM2G095806	B8A1T6	HPPK/DHPS	HPPK/DHPS	
GRMZM2G416386	K7VD39	ADCS	ADCS	
GRMZM2G108416	B6TME5	ADCL	ADCL1	ADCL1 and ADCL2: 45.3 %
GRMZM2G087103	A0A096R079	ADCL	ADCL2	ADCL1 and ADCL3: 46.6 %
GRMZM2G069596	A0A096RBT2	ADCL	ADCL3	ADCL2 and ADCL3: 71.0 %
GRMZM2G304915	K7TY68	DHFS	DHFS-1	DHFS-1 and DHFS-2: 92.7 %
GRMZM2G169481	A0A096SVY8	DHFS	DHFS-2	
GRMZM2G072608	K7TWH4	DHFR	DRTS-1	DRTS-1 and DRTS-2: 97.3 %; DRTS-1 and DRTS-3: 92.2 %
GRMZM2G421493	A0A096TQ18	DHFR	DRTS-2	DRTS-1 and DRTS-4: 95.4 %; DRTS-2 and DRTS-3: 90.8 %
GRMZM2G005990	O81395	DHFR	DRTS-3	DRTS-2 and DRTS-4: 95.9 %; DRTS-3 and DRTS-4: 97.7 %
GRMZM2G139880	K7UAA2	DHFR	DRTS-4	
GRMZM5G869779	A0A096UEV9	FPGS	FPGS-1	FPGS-1 and FPGS-2: 77.8 %
GRMZM2G393334	K7VM84	FPGS	FPGS-2	

Note: All accession numbers were obtained from www.uniprot.org [38]

Eight enzymes involved in C1 metabolism in maize were also identified, which were annotated as SHMT, GDC complex (GDCH, GDCP, and GDCT), DHC, MTHFR, MS, 10-FDF, FTHS, and 5-FCL, respectively. Because SHMT1 is the major functional SHMT enzyme in Arabidopsis [13, 14], maize SHMT1, the closest counterpart of Arabidopsis SHMT1, was used in this study. We found that the maize GDC protein complex consisted of one GDCP, one GDCT, and four GDCHs, and the lowest sequence similarity to maize GDCH among the GDCH orthologs was 71.2 %. 10-FDF and FTHS each had one ortholog; MTHFR and 5-FCL each had two orthologs, and the sequence similarity between each pair of orthologs was 94.5 % and 51.2 %, respectively. DHC and MS each had three orthologs, and the lowest sequence similarities among orthologs were 61.0 % (between FOLD2 and FOLD3) and 96.3 % (between MS1 and MS2), respectively (Table 2). These results indicated that the majority of orthologs involved in C1 metabolism at protein level were highly conserved in maize.

**Table 2:** Genes involved in C1 metabolism in maize

Gene identifier	Accession number	Gene function	Protein abbreviation	Sequence similarity among orthologs
GRMZM2G135283	B6T7Q7	SHMT1	SHMT1	
GRMZM2G399183	K7UCR4	GDCH	GCSH1	GCSH1 and GCSH2: 72.3 %; GCSH1 and GCSH3: 71.2 %
GRMZM2G010321	B4FUR6	GDCH	GCSH2	GCSH1 and GCSH4: 73.1 %; GCSH2 and GCSH3: 96.2 %
GRMZM2G051208	C4JBL9	GDCH	GCSH3	GCSH2 and GCSH4: 93.8 %; GCSH3 and GCSH4: 94.8 %
GRMZM2G020288	K7TZ76	GDCH	GCSH4	
GRMZM2G104310	K7TX08	GDCP	GCSP	
GRMZM5G876898	B6TQ06	GDCT	GCST	
GRMZM2G130790	C4JC05	DHC	FOLD1	FOLD1 and FOLD2: 66.6 %
GRMZM2G150485	K7UXQ3	DHC	FOLD2	FOLD1 and FOLD3: 67.6 %
AC233922.1_FG005	B7ZXD5	DHC	FOLD3	FOLD2 and FOLD3: 61.0 %
GRMZM2G347056	NP_001104947	MTHFR	MTHR1	MTHR1 and MTHR2: 94.5 %
GRMZM2G034278	A0A096QBQ5	MTHFR	MTHR2	
GRMZM2G149751	A0A096SHX7	MS	MS1	MS1 and MS2: 96.3 %
GRMZM2G112149	A0A096RTH2	MS	MS2	MS1 and MS3: 96.7 %
GRMZM2G165747	B6UF55	MS	MS3	MS2 and MS3: 99.0 %
GRMZM2G168281	K7WHT7	10-FDF	PURU	
GRMZM5G824944	A0A096U8U8	FTHS	FTHS	
GRMZM5G807835	A0A096U6Q0	5-FCL	5FCL	5FCL and 5FCLL: 51.2 %
GRMZM2G001904	K7TIY8	5-FCL	5FCLL	

Note: All accession numbers were obtained from www.uniprot.org [38], with the exception of the accession number of MTHR1, which was from http://www.ncbi.nlm.nih.gov [36]

To investigate whether folate metabolism-related proteins identified in maize contain conserved domains for their enzymatic activities, all homologs from plants (*e.g.* sorghum, rice, millet, and Arabidopsis), mammals (*e.g.* human, rat and mouse), and microorganisms (*e.g.* yeast and *E. coli*) were analyzed using Simple Modular Architecture Research Tool [15] (SMART). As expected, the enzymes participating in folate metabolism and C1 cycle were largely conserved between maize and other species. The representative proteins from maize, Arabidopsis, and *E. coli* are shown in Tables 3 and 4. A detailed comparison of the enzymes involved in folate synthesis between the three species led to the following interesting findings. First, the same PFAM domains were present with different lengths. For example, both FPGS and DHFS contained the Mur_ligase_M domain that is responsible for attaching glutamates to folylpolyglutamates or monoglutamates, respectively. However, the Mur_ligase_M domain in FPGS was 36-amino acid shorter than that in DHFS both in maize and Arabidopsis (Table 3)*.* Second, GTPCHI evolved two repeats of the GTP_cyclohydroI domain in the plants, while only one in *E. coli* (Table 3). Third, three enzymes, including ADCS, HPPK/DHPS, and DHFR/TS, have evolved to be bifunctional enzymes in the plants. For example, both maize and Arabidopsis ADCS contained two GATases, one Anth_synt_I_N, and one chorismate_binding domain, functionally corresponding to Anth_synt_I_N and chorismate_binding-containing PABA and GATase-containing PABB in *E. coli* to produce ADC. Similar phenomena were observed in HPPK/DHPS and DHFR/TS, respectively (Table 3). Two enzymes involved in C1 reactions contained different number of PFAM domains in different species. For example, three GCV_T domains were present in the maize GCST, whereas two in Arabidopsis and *E. coli*. The five domains in *E. coli* MS, i.e. S-methyl_trans, Pterin_bind, B12-binding, B12-binding_2, and Met_synt_B12, were found to be merged as two domains of Meth_synt_1 and Meth_synt_2 in Arabidopsis and maize (Table 4).

**Table 3:** Conserved domains in enzymes of folate synthesis in maize, Arabidopsis, and *E. coli*

Enzymes	Domain numbers	Domain names	Domain size in AA	Enzymes	Domain numbers	Domain names	Domain size in AA
ZmGCH1-1	2	GTP_cyclohydroI	190	ZmHPPK/DHPS	2	HPPK	125
		GTP_cyclohydroI	189			Pterin_bind	220
ZmGCH1-2	2	GTP_cyclohydroI	193	AtHPPK/DHPS1	2	HPPK	125
		GTP_cyclohydroI	192			Pterin_bind	220
AtGCH1	2	GTP_cyclohydroI	156	AtHPPK/DHPS2	2	HPPK	126
		GTP_cyclohydroI	183			Pterin_bind	220
EcGCH1	1	GTP_cyclohydroI	179	EcHPPK	1	HPPK	127
ZmFOLB1	1	FolB	113	EcDHPS	1	Pterin_bind	205
ZmFOLB2	1	FolB	113	ZmDHFS-1	1	Mur_ligase_M	246
AtFOLB1	1	FolB	114	ZmDHFS-2	1	Mur_ligase_M	245
AtFOLB2	1	FolB	114	AtDHFS	1	Mur_ligase_M	244
AtFOLB3	1	FolB	114	EcFOLC	2	Mur_ligase_M	214
EcFOLB	1	FolB	122			Mur_ligase_C	80
ZmADCS	4	GATase	174	ZmDRTS-1	2	DHFR_1	168
		GATase	61			Thymidylat_synt	82
		Anth_synt_I_N	153	ZmDRTS-2	2	DHFR_1	176
		Chorismate_bind	258			Thymidylat_synt	82
AtADCS	4	GATase	171	ZmDRTS-3	2	DHFR_1	177
		GATase	58			Thymidylat_synt	283
		Anth_synt_I_N	155	ZmDRTS-4	2	DHFR_1	177
		Chorismate_bind	258			Thymidylat_synt	283
EcPABB	2	Anth_synt_I_N	138	AtDRTS1	2	DHFR_1	177
		Chorismate_bind	254			Thymidylat_synt	283
EcPABA	1	GATase	184	AtDRTS2	2	DHFR_1	177
ZmADCL1	1	Aminotran_4	235			Thymidylat_synt	283
ZmADCL2	1	Aminotran_4	239	AtDRTS3	2	DHFR_1	177
ZmADCL3	1	Aminotran_4	235			Thymidylat_synt	257
AtADCL1	1	Aminotran_4	235	EcDYR	1	DHFR_1	152
AtADCL2	1	Aminotran_4	235	EcTYSY	1	Thymidylat_synt	263
AtADCL3	1	Aminotran_4	236	ZmFPGS-1	1	Mur_ligase_M	210
EcPABC	1	Aminotran_4	229	ZmFPGS-2	1	Mur_ligase_M	209
				AtFPGS1	1	Mur_ligase_M	209
				AtFPGS2	1	Mur_ligase_M	209
				AtFPGS3	1	Mur_ligase_M	205
				EcFOLC	2	Mur_ligase_M	214
						Mur_ligase_C	80

Note: All domain information was extracted from http://smart.embl-heidelberg.de/ [15]

AA represents amino acid

**Table 4:** Conserved domains in enzymes of C1 metabolism in maize, Arabidopsis, and *E. coli*

Enzymes	Domain numbers	Domain names	Domain size in AA	Enzymes	Domain numbers	Domain names	Domain size in AA
ZmSHMT1	1	SHMT	398	ZmMTHR1	1	MTHFR	297
AtSHMT1	1	SHMT	398	ZmMTHR2	1	MTHFR	266
EcGLYA	1	SHMT	378	AtMTHR1	1	MTHFR	295
ZmGCSH1	1	GCV_H	120	AtMTHR2	1	MTHFR	295
ZmGCSH2	1	GCV_H	120	EcMETF	1	MTHFR	280
ZmGCSH3	1	GCV_H	120	ZmMS1	2	Meth_synt_1	317
ZmGCSH4	1	GCV_H	99			Meth_synt_2	324
AtGCSH1	1	GCV_H	120	ZmMS2	2	Meth_synt_1	316
AtGCSH2	1	GCV_H	120			Meth_synt_2	324
AtGCSH3	1	GCV_H	120	ZmMS3	2	Meth_synt_1	316
EcGCSH	1	GCV_H	121			Meth_synt_2	324
ZmGCSP	2	GDC-P	428	AtMS1	2	Meth_synt_1	316
		GDC-P	291			Meth_synt_2	324
AtGCSP1	2	GDC-P	427	AtMS2	2	Meth_synt_1	316
		GDC-P	288			Meth_synt_2	324
AtGCSP2	2	GDC-P	428	AtMS3	2	Meth_synt_1	316
		GDC-P	290			Meth_synt_2	324
EcGCSP	2	GDC-P	424	EcMETH	5	S-methyl_trans	311
		GDC-P	354			Pterin_bind	212
ZmGCST	3	GCV_T	152			B12-binding	104
		GCV_T_C	92			B12-binding_2	83
		GCV_T	89			Met_synt_B12	273
AtGCST	2	GCV_T	215	ZmFTHS	1	FTHFS	620
		GCV_T_C	92	AtFTHS	1	FTHFS	620
EcGCST	2	GCV_T	208	ZmPURU	1	Formyl_trans_N	178
		GCV_T_C	92	AtPURU1	1	Formyl_trans_N	178
ZmFOLD1	2	THF_DHG_CYH	117	AtPURU2	1	Formyl_trans_N	178
		THF_DHG_CYH_C	167	EcPURU	1	Formyl_trans_N	177
ZmFOLD2	2	THF_DHG_CYH	117	Zm5FCL	1	5-FTHF_cyc-lig	205
		THF_DHG_CYH_C	105	At5FCL	1	5-FTHF_cyc-lig	203
ZmFOLD3	2	THF_DHG_CYH	117	Ec5FCL	1	5-FTHF_cyc-lig	175
		THF_DHG_CYH_C	167	Zm5FCLL	1	5-FTHF_cyc-lig	101
AtFOLD1	2	THF_DHG_CYH	117	At5FCLL	1	5-FTHF_cyc-lig	198
		THF_DHG_CYH_C	167				
AtFOLD2	2	THF_DHG_CYH	117				
		THF_DHG_CYH_C	167				
AtFOLD3	2	THF_DHG_CYH	53				
		THF_DHG_CYH_C	167				
AtFOLD4	2	THF_DHG_CYH	117				
		THF_DHG_CYH_C	167				
EcFOLD	2	THF_DHG_CYH	117				
		THF_DHG_CYH_C	159				

Note: All domain information was extracted from http://smart.embl-heidelberg.de/ [15]

AA represents amino acid

**Fig. 2:**
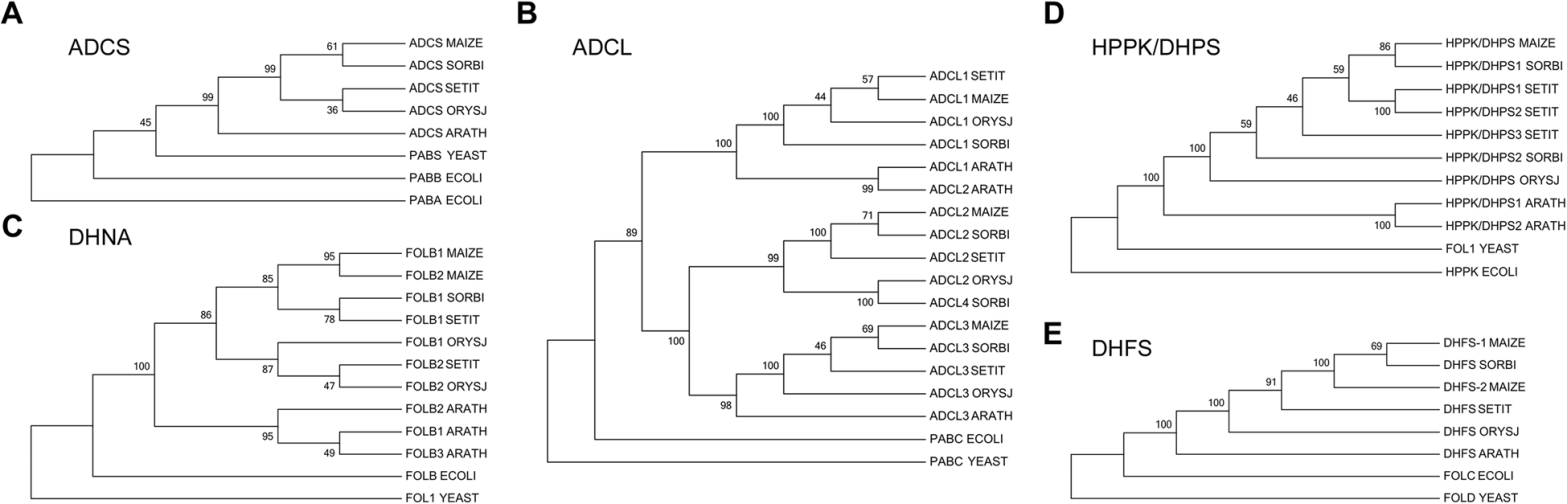
Phylogenetic trees of folate-metabolism related proteins which lack homologs in animals. Phylogenetic trees of folate-metabolism related proteins (which lack homologs in animals) from maize, sorghum, millet, rice, Arabidopsis, yeast, and *E. coli* constructed by MEGA version 5 using neighbour-joining algorithms. **a**, ADCS; **b**, ADCL; **c**, DHNA; **d**, HPPK/DHPS; **e**, DHFS. Accession numbers used in this figure are: ADCS SORBI (Swiss-Prot: C5Z8W2), ADCS SETIT (K3XV74), ADCS ORYSJ (Q5Z856), ADCS ARATH (Q8LPN3), PABA ECOLI (P00903), PABB ECOLI (P05041), PABS YEAST (P37254); ADCL2 SORBI (C5XJI9), ADCL3 SORBI (C5XZZ4); ADCL4 SORBI (C5YVA1), ADCL1 SETIT (K4A646), ADCL2 SETIT (K3XJT1), ADCL3 SETIT (K3YT16); ADCL1 ORYSJ (Q10L48), ADCL2 ORYSJ (Q5W706), ADCL3 ORYSJ (B8AFD4); ADCL1 ARATH (Q8W0Z7), ADCL2 ARATH (Q9ASR4), ADCL3 ARATH (Q8L493), PABC ECOLI (P28305), PABC YEAST (Q03266); FOLB1 SORBI (C5YNA8), FOLB1 SETIT(K3YK60), FOLB2 SETIT (K3ZWK7), FOLB2 ORYSJ (Q653D9),FOLB1 ARATH (A2RVT4), FOLB2 ARATH (Q9FM54), FOLB3 ARATH (Q6GKX5), FOLB ECOLI (P0AC16),FOL1 YEAST (P53848); HPPK/DHPS2 SORBI (C5XIR9), HPPK/DHPS1 SORBI (C5X2E7), HPPK/DHPS1 SETIT (K3XGF0), HPPK/DHPS2 SETIT (K3ZID4), HPPK/DHPS3 SETIT (K3ZSW5), HPPK/DHPS ORYSJ (Q7X7X0),HPPK/DHPS2 ARATH (Q1ENB6), HPPK/DHPS1 ARATH (F4JPH1), HPPK ECOLI (P26281), FOL1 YEAST (P53848); DHFS SORBI (C5YPL9),DHFS SETIT (K3ZS10), DHFS ORYSJ (Q2QLY6), DHFS ARATH (F4JYE9), FOLC ECOLI (P08192), FOLD YEAST (Q12676); ADCL1 SORBI (Phytozome: Sb01g034820.1), and FOLB1 ORYSJ (LOC_Os06g06100.1)

**Fig. 3:**
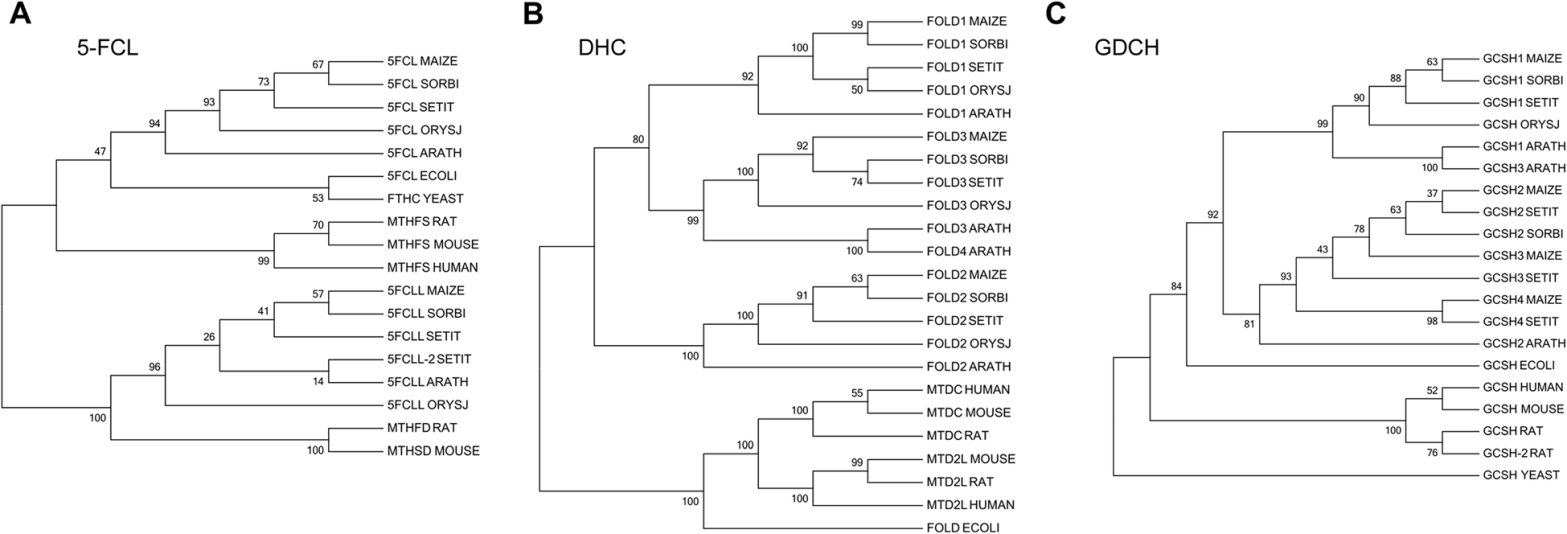
Phylogenetic trees of 5-FCL, DHC, and GDCH proteins. Phylogenetic trees of 5-FCL, DHC, and GDCH proteins from maize, sorghum, millet, rice, Arabidopsis, human, rat, mouse, yeast, and *E. coli* constructed by MEGA version 5 using neighbour-joining algorithms. Plant branches are divided into multiple classes. **a**, 5-FCL; **b**, DHC; **c**, GDCH. The accession numbers are: 5FCL SORBI (Swiss-Prot: C5XCF3), 5FCLL SORBI (C5YSM0), 5FCLL SETIT (K3Y8D4), 5FCL SETIT (K3ZVU5), 5FCLL-2 SETIT (K3YF41), 5FCL ORYSJ (Q0D564), 5FCLL ORYSJ (Q2QX67); 5FCL ARATH (Q8L539), 5FCLL ARATH (Q9SRE0), 5FCL ECOLI (P0AC28), FTHC YEAST (P40099), MTHFS HUMAN (P49914), MTHFS RAT (Q5M9F6), MTHFD RAT (M0R5E8), MTHSD MOUSE (Q3URQ7), MTHFS MOUSE (Q9D110); FOLD1 SORBI (C5X9V9), FOLD2 SORBI (C5Z052), FOLD3 SORBI (C5XT02), FOLD1 SETIT (K3ZU46), FOLD2 SETIT (K3Z8H6), FOLD3 SETIT (K3YTG4), FOLD1 ORYSJ (Q6K2P4), FOLD2 ORYSJ (B9FHE0), FOLD3 ORYSJ (Q0E4G1), FOLD1 ARATH (A2RVV7), FOLD2 ARATH (Q9LHH7), FOLD3 ARATH (O65269), FOLD4 ARATH (O65271), FOLD ECOLI (P24186), MTD2L HUMAN (Q9H903), MTDC HUMAN (P13995), MTD2L RAT (D3ZUA0), MTDC RAT (D4A1Y5), MTDC MOUSE (P18155), MTD2L MOUSE (D3YZG8); GCSH1 SORBI (C5YT80), GCSH2 SORBI (C5XW40), GCSH1 SETIT (K3YAF8), GCSH2 SETIT (K3YWB1), GCSH3 SETIT (K3ZA97), GCSH4 SETIT (K3YMG1), GCSH ORYSJ (A3C6G9), GCSH1 ARATH (P25855), GCSH2 ARATH (O82179), GCSH3 ARATH (Q9LQL0), GCSH ECOLI (P0A6T9), GCSH YEAST (P39726), GCSH HUMAN (P23434), GCSH RAT (Q5I0P2), GCSH-2 RAT (Q9QYU8), and GCSH MOUSE (Q91WK5)

**Fig. 4:**
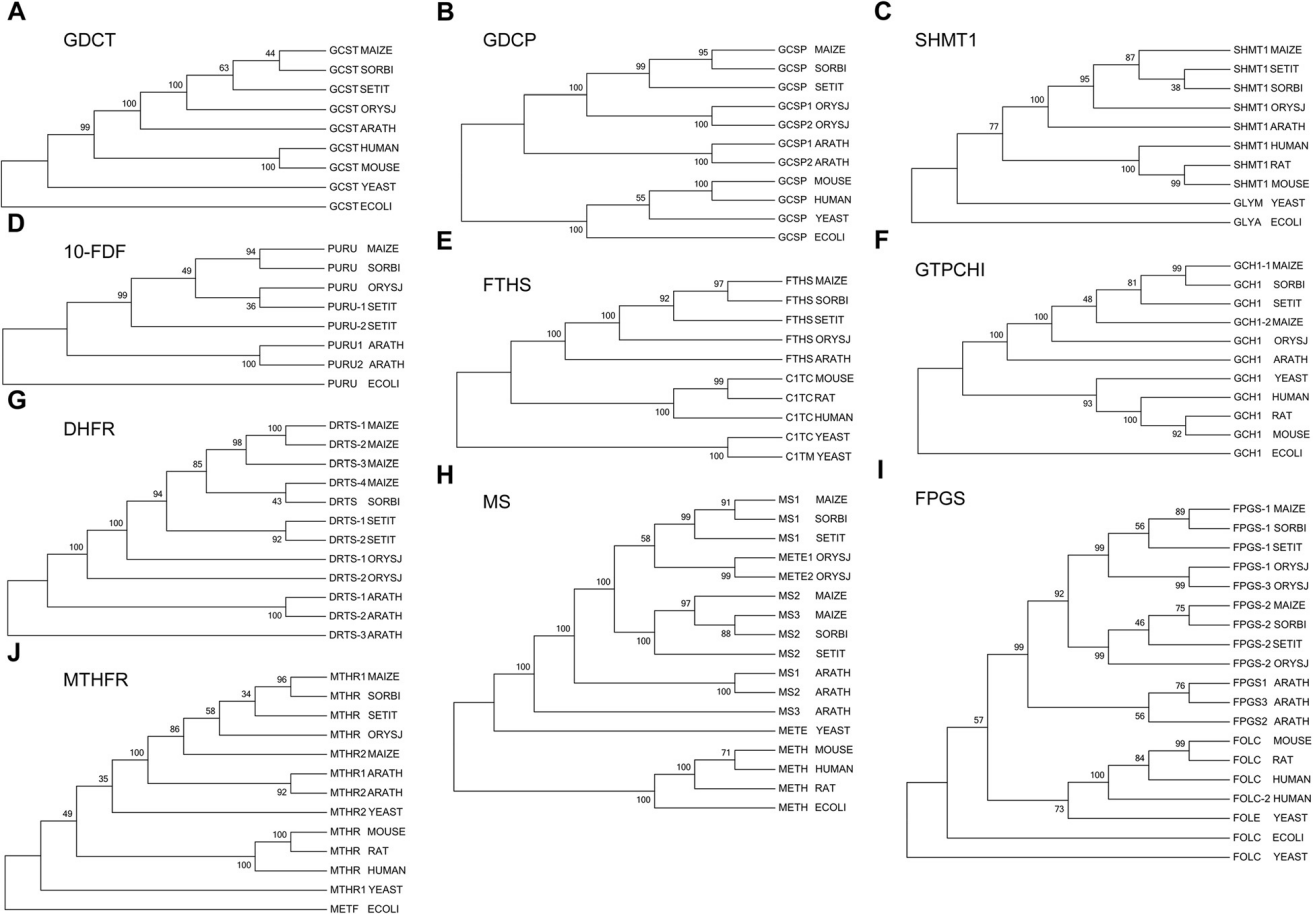
Phylogenetic trees of folate-metabolism related proteins which all plant homologs are grouped into one class. The phylogenetic trees of folate-metabolism related proteins from maize, sorghum, millet, rice, Arabidopsis, human, rat, mouse, yeast, and *E. coli* constructed by MEGA version 5 using neighbour-joining algorithms. All plant homologs are grouped into one class. **a**, GDCT; **b**, GDCP; **c**, SHMT1; **d**, 10-FDF; **e**, FTHS; **f**, GTPCHI; **g**, DHFR; **h**, MS; **i**, FPGS; **j**, MTHFR. The accession numbers used in this figure are: GCST SORBI (Swiss-Prot: C5YG66), GCST SETIT (K3Y7N9), GCST ORYSJ (Q01KC0), GCST ARATH (O65396), GCST ECOLI (P27248), GCST YEAST (P48015), GCST HUMAN (P48728), GCST MOUSE (Q8CFA2); GCSP SORBI (C5YS41), GCSP SETIT (K3XDV1), GCSP1 ORYSJ (Q6RS61), GCSP2 ORYSJ (Q6V9T1), GCSP1 ARATH (Q94B78), GCSP2 ARATH (O80988), GCSP ECOLI (P33195), GCSP YEAST (P49095), GCSP HUMAN (P23378), GCSP MOUSE (Q91W43); SHMT1 SETIT (K4A8N1), SHMT1 ORYSJ (Q10D68), SHMT1 ARATH (Q9SZJ5), GLYA ECOLI (P0A825), GLYM YEAST (P37292), SHMT1 HUMAN (P34896), SHMT1 RAT (Q6TXG7), SHMT1 MOUSE (P50431); PURU SORBI (C5WMW1), PURU-1 SETIT (K4ACX9), PURU-2 SETIT (K3Z0D3), PURU ORYSJ (Q10T42), PURU1 ARATH (Q93YQ3), PURU2 ARATH (F4JP46), PURU ECOLI (P37051); FTHS SORBI (C5X255), FTHS SETIT (K3ZR21), FTHS ORYSJ (Q0J1E1), FTHS ARATH (Q9SPK5), CITC YEAST (P07245), C1TM YEAST (P09440), C1TC HUMAN (P11586), C1TC RAT (P27653), C1TC MOUSE (Q922D8); GCH1 SETIT (K3Z5X1), GCH1 ARATH (Q9SFV7), GCH1 ECOLI (P0A6T5), GCH1 YEAST (P51601), GCH1 HUMAN (P30793), GCH1 RAT (P22288), GCH1 MOUSE (Q05915); DRTS SORBI (C5Y2E9), DRTS-1 SETIT (K3ZI20), DRTS-2 SETIT (K3ZSB7), DRTS-1 ORYSJ (Q2R481), DRTS-2 ORYSJ (Q2QRX6), DRTS-1 ARATH (Q05762), DRTS-2 ARATH (Q05763), DRTS-3 ARATH (Q9SIK4); MS2 SORBI (Q8W0Q7), MS1 SETIT (K3Z414), MS2 SETIT (K4A622), METE1 ORYSJ (Q2QLY5), METE2 ORYSJ (Q2QLY4), MS1 ARATH (O50008), MS2 ARATH (Q9SRV5), MS3 ARATH (Q0WNZ5), METH ECOLI (P13009), METE YEAST (P05694), METH HUMAN (Q99707), METH RAT (Q9Z2Q4), METH MOUSE (A6H5Y3); FPGS-1 SORBI (C5WWE5), FPGS-2 SORBI (C5WMM8), FPGS-1 SETIT (K4A7H2), FPGS-2 SEITI (K4A839), FPGS-1 ORYSJ (Q337F3), FPGS-2 ORYSJ (Q10SU1), FPGS-3 ORYSJ (B9G6I2), FPGS1 ARATH (F4K2A1), FPGS2 ARATH (F4J2K2), FPGS3 ARATH (Q8W035), FOLC ECOLI (P08192), FOLE YEAST (Q08645), FOLC YEAST (P36001), FOLC HUMAN (Q05932), FOLC-2 HUMAN (Q5JU23), FOLC RAT (M0R401), FOLC MOUSE (P48760); MTHR SORBI (C5WVY7), MTHR SETIT (K4AMY6), MTHR ORYSJ (Q75HE6), MTHR1 ARATH (Q9SE60), MTHR2 ARATH (O80585), METF ECOLI (P0AEZ1), MTHR1 YEAST (P46151), MTHR2 YEAST (P53128), MTHR HUMAN (P42898), MTHR RAT (D4A7E8), MTHR MOUSE (Q9WU20); SHMT1 SORBI (phytozome: Sb01g008690.1), GCH1 SORBI (Sb06g031800.1), GCH1 ORYSJ (LOC_Os04g56710.1), and MS1 SORBI (Sb08g022210.1)

Phylogenetic trees of folate-related proteins from sorghum, rice, millet, Arabidopsis, human, rat, mouse, yeast and *E. coli* were constructed using the neighbour-joining method. The majority of clade credibility values between maize and sorghum or millet were higher than 70 %, suggestive of a close relationship between the enzymes in maize with those in sorghum and millet. These observations are consistent with the fact that maize, sorghum, and millet share a common C4 origin [16, 17] (Figs. 2, 3, 4). Some homologs, including ADCS, ADCL, DHNA, HPPK/DHPS, and DHFS, were not present in animals (Fig. 2), and the remaining homologs from plants and animals were divided into two sibling groups (Figs. 3 and 4). There was a special type of tree where the plant branches were divided into multiple classes, and each class contained most of the plant species, such as DHC, ADCL, 5-FCL, and GDCH (Table 1 and Table 2). The remaining trees were characterized that all the plant homologs were classed as a single clade, in which the maize orthologs were either present as a single gene, such as ADCS, HPPK/DHPS, GDCT, GDCP, SHMT1, HPPK/DHPS, 10-FDF, and FTHS, or as multiple genes, such as DHNA, DHFS, GTPCHI, DHNA, DHFS, DHFR, MS, FPGS, and MTHFR (Figs. 2, 3, 4; Table 1 and Table 2). These results indicate that the folate metabolism-related proteins are conserved in maize, and the differentiation of the function of these proteins is complicated during the evolutionary process.

Maize differed from Arabidopsis in the number of genes participating in folate and C1 metabolism. For example, more orthologs of DHFR, GTPCHI, DHFS, and GDCH as well as less orthologs of DHNA, 10-FDF, FPGS, DHC, HPPK/DHPS, and GDCP were identified in maize than in Arabidopsis. Of these enzymes, four, including AtDHFS, AtFPGS1, AtFPGS2, and AtFPGS3, functioned as a ligase in Arabidopsis [18] (Table 2). A mutation in AtDHFS caused embryo lethality [19], and the dysfunction of FPGS1 or FPGS2 resulted in abnormal responses to low nitrogen in the dark or light [20, 21]. These reports are suggestive of distinct functions between the DHFS and FPGS in Arabidopsis, albeit they contain the same domain. In maize, the Mur_ligase_M domain was also found to be present in the corresponding orthologs, including two DHFSs and two FPGSs, and further biochemical and genetic studies on these orthologs will elucidate their biological functions.

DHNAs were reported to have distinct expression pattern between Arabidopsis and maize [22, 23]. In Arabidopsis, three DHNA orthologs were identified, among which *AtFolB2* was highly expressed in roots, stems, siliques, young leaves, and mature leaves, whereas *AtFolB3* was undetectable [22]. However, only two DHNA orthologs were identified (Fig. 2). The transcripts of *FOLB1 MAIZE* and *FOLB2 MAIZE* were abundant in roots, shoots, developing leaves and tassels, and seeds [23]. These observations imply that the maize orthologs may play different roles than Arabidopsis ones.

### Folate profiling in maize kernels

Maize kernels are the primary source of folates for humans [24]. Investigation of folate biosynthesis during kernel formation and in mature seeds is important for understanding folate metabolic flux in maize. To this end, two representative maize inbred lines with a significant difference in total folates in dry seeds were chosen. Ji63 is originated from China, belonging to the NSS subpopulation with pedigree being (127-32 × Tie84) × (Wei24 × Wei20); GEMS31 is from the United States, belonging to the TST subpopulation with pedigree being 2282-01_XL380_S11_F2S4_9226-Blk26/00 [25]. 5-F-THF and 5-M-THF in the dry seeds from these two inbred lines grown in different locations were measured using liquid chromatography-tandem mass spectroscopy (LC/MS). Irrespective of the significant variations across the years, GEMS31 contained a lot more total folates than Ji63, with 12.9 folds being the smallest difference in 2010 (Table 5). Moreover, it was observed that 5-F-THF accounted for over 70.3 % of total folates in Ji63 and 94.4 % in GEMS31 across the four consecutive years. These results indicated that 5-F-THF was the major storage form of folate derivative in both GEMS31 and Ji63 regardless of the total folate levels in dry seeds.

**Table 5:** The contents of total folate and the proportion of 5-F-THF in mature dry seeds

Total folates (nmol/g DW)					The proportion of 5-F-THF (%)	
Year	Location	GEMS31	Ji63	GEMS31/Ji63	GEMS31	Ji63
2009	Hainan, China	18.89	1.24	15.2	94.4	87.1
2010	Yunnan, China	8.25	0.64	12.9	97.1	70.3
2012	Hainan, China	3.96	0.26	15.2	95.7	80.8
2013	Beijing, China	5.45	0.27	20.2	96.7	74.1

Note: Total folates contain 5-F-THF and 5-M-THF

Each inbred line was measured once across the four consecutive years

**Fig. 5:**
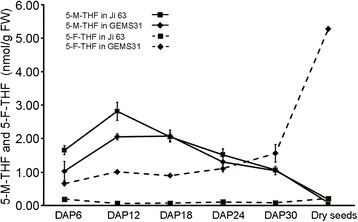
Folate profiling of kernels during formation. Folate profiling of kernels during formation and in dry seeds of Ji63 and GEMS31, respectively. Data are means ± SD (n = 4), and each replicate consisted of 50 mg of plant material. DAP, days after pollination

To investigate how folate derivatives are accumulated during kernel formation, the kernels at R1 (silking stage) on DAP 6, R2 (blistering stage) on DAP 12, R3 (milking stage) on DAP 18, R4 (late milk-dough stage) on DAP 24, and R5 (early dent stage) on DAP 30 were collected for LC-MS analysis in 2013. In contrast to that in dry seeds, 5-M-THF was more accumulated than 5-F-THF in young seeds of both lines from DAP 6 to DAP18. GEMS31 and Ji63 contained similar levels of total folates in the seeds at the early developmental stages which was indicated by the ratio of folates in GEMS31 vs folates in Ji 63 being around 1 (0.91 on DAP 6 and 1.07 on DAP 12). At the late developmental stages, i.e. DAP 18 and DAP 30, the total folates in GEMS31 were significantly higher than that in Ji63 from (Fig. 5). These results were quite different from that observed in dry seeds, suggesting an ongoing active folate metabolism during the seed maturation.

5-M-THF accounted for over 60 % of the total folates in GEMS31 (61.1 % for DAP 6, 67.2 % for DAP 12, and 69.9 % for DAP 18) and over 90.2 % in Ji63 (90.2 % for DAP 6, 98.3 % for DAP 12, and 97.1 % for DAP 18) during early stages of kernel formation (Table 6). However, no significant change in 5-F-THF was observed before DAP 18 in either of the inbred lines: 5-F-THF in GEMS31 maintained ~0.80 nmol/g FW, while that in Ji63 ~ 0.10 nmol/g FW before DAP18. After DAP 18, 5-M-THF was decreased to a similar level in both lines, and the proportion of 5-M-THF was also reduced due to the increased 5-F-THF (Fig. 5; Table 6). Notably, from DAP 30 on, a much sharper increase of 5-F-THF was observed in GEMS31 than in Ji63 (Fig. 5). The profiling of these two inbred lines demonstrated that 5-M-THF was the dominant folate derivative at least before DAP 18, implying a more active C1 reaction at early stages of seed development than late stages given the fact that 5-M-THF is the donor for C1 cycle.

**Table 6:** The contents of total folate and proportion of 5-M-THF during the early stage of kernel formation

Total folates (nmol/g FW)					The proportion of 5-M-THF (%)		
DAP	GEMS31	Ji63	GEMS31/Ji63	*T*-test	GEMS31	Ji63	*T*-test
DAP 6	1.67 ± 0.35	1.83 ± 0.15	0.91	0.399	61.1	90.2	7.48E-07
DAP 12	3.05 ± 0.12	2.86 ± 0.27	1.07	0.256	67.2	98.3	4.11E-09
DAP 18	2.89 ± 0.09	2.10 ± 0.13	1.41	0.850	69.9	97.1	5.66E-08
DAP 24	2.39 ± 0.25	1.61 ± 0.18	1.48	0.008	54.1	93.9	7.81E-07
DAP 30	2.59 ± 0.38	1.13 ± 0.02	2.30	0.003	40.0	93.4	2.30E-06

Note: DAP, days after pollination

Total folates contain 5-F-THF and 5-M-THF

Data are means ± SD (n = 4), and each replicate consisted of 50 mg of plant material

Different metabolites show different accumulation patterns during seed development, and the storage metabolites normally start to accumulate from the early developmental stage [26, 27]. In maize, over 80 % of total starch is stored in the endosperm, 80 % of total oil in the embryo, and proteins are found in both the embryo and endosperm [28]. The rate of oil synthesis typically peaks between DAP 15 and DAP 25, and the accumulation peaks on DAP 30; carotenoids behave in a similar manner [29]. Starch accumulation occurs from DAP 10, peaks on DAP 15, and remains steady thereafter [27]. Likewise, amino acids accumulate during the early stage, and steady-state transcripts of the genes involved in amino acid biosynthesis peak in kernels on DAP 10 and in embryos on DAP 15 [26]. It has also been reported that some metabolites are decreased during kernel formation. For example, flavone is decreased during DAP 14 to DAP 40 in maize [30]. Unlike the metabolites mentioned above, folate derivatives showed different accumulation patterns in maize kernels. 5-M-THF peaked on DAP 12 and consistently decreased, whereas 5-F-THF remained unchanged at low levels during the early stages, but gradually increased to high levels in dry seeds (Fig. 5). These results indicate that the various folate derivatives may differ one aother in functioning during seed development in maize.

### Transcript expression of folate-related genes in maize kernel

**Fig. 6:**
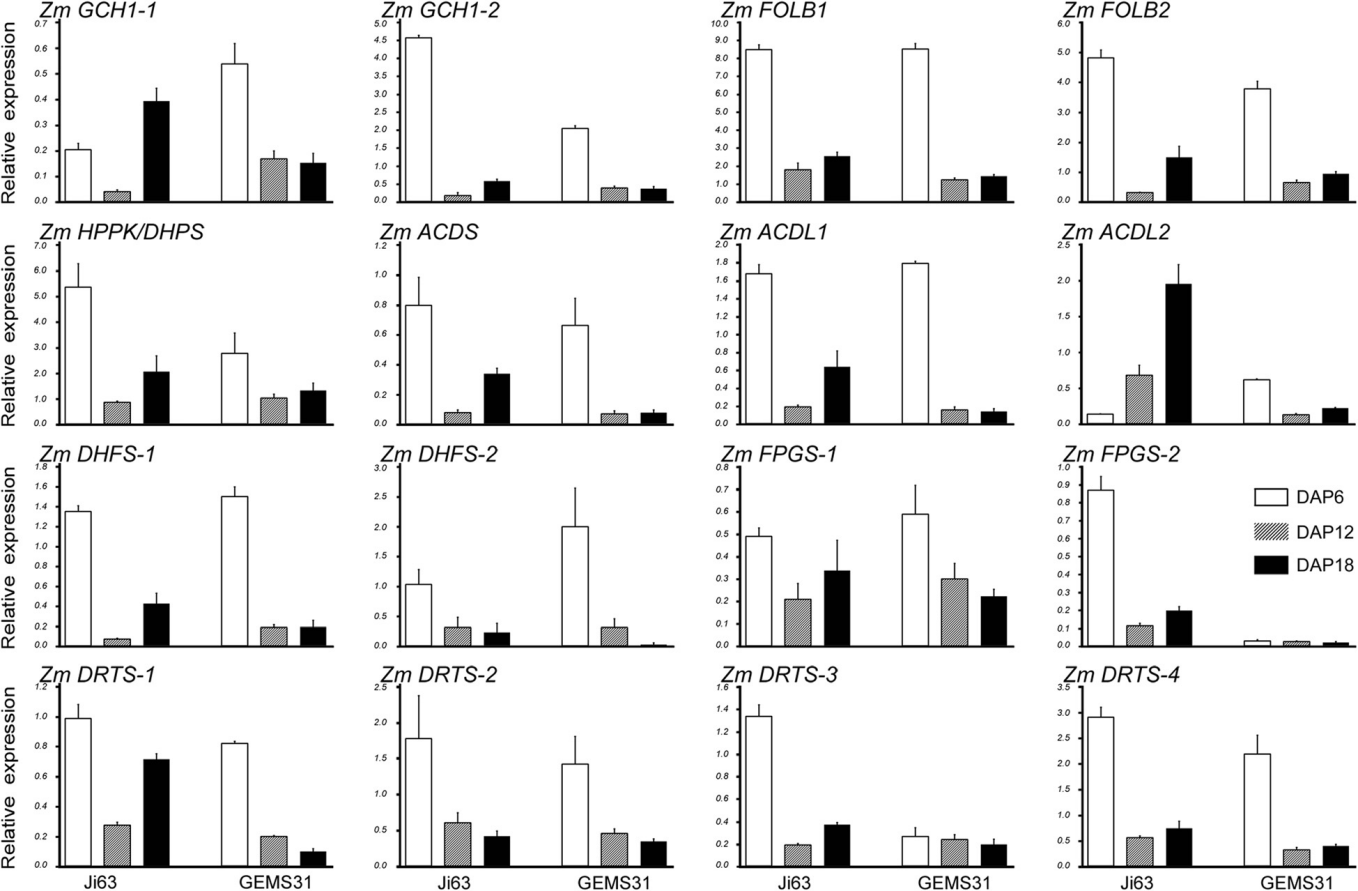
qRT-PCR of folate-synthesis related genes during kernel formation. qRT-PCR of folate-synthesis related genes during kernel formation of Ji63 and GEMS31, respectively. Three biological samples were used for analysis and all reactions were performed in quadruplicate. Data are means ± SD (n = 4). Names of the proteins are listed in Table 1. The same samples were used as that used for folate profiling. Because expression of *ADCL3* was not detected, it’s not shown

**Fig. 7:**
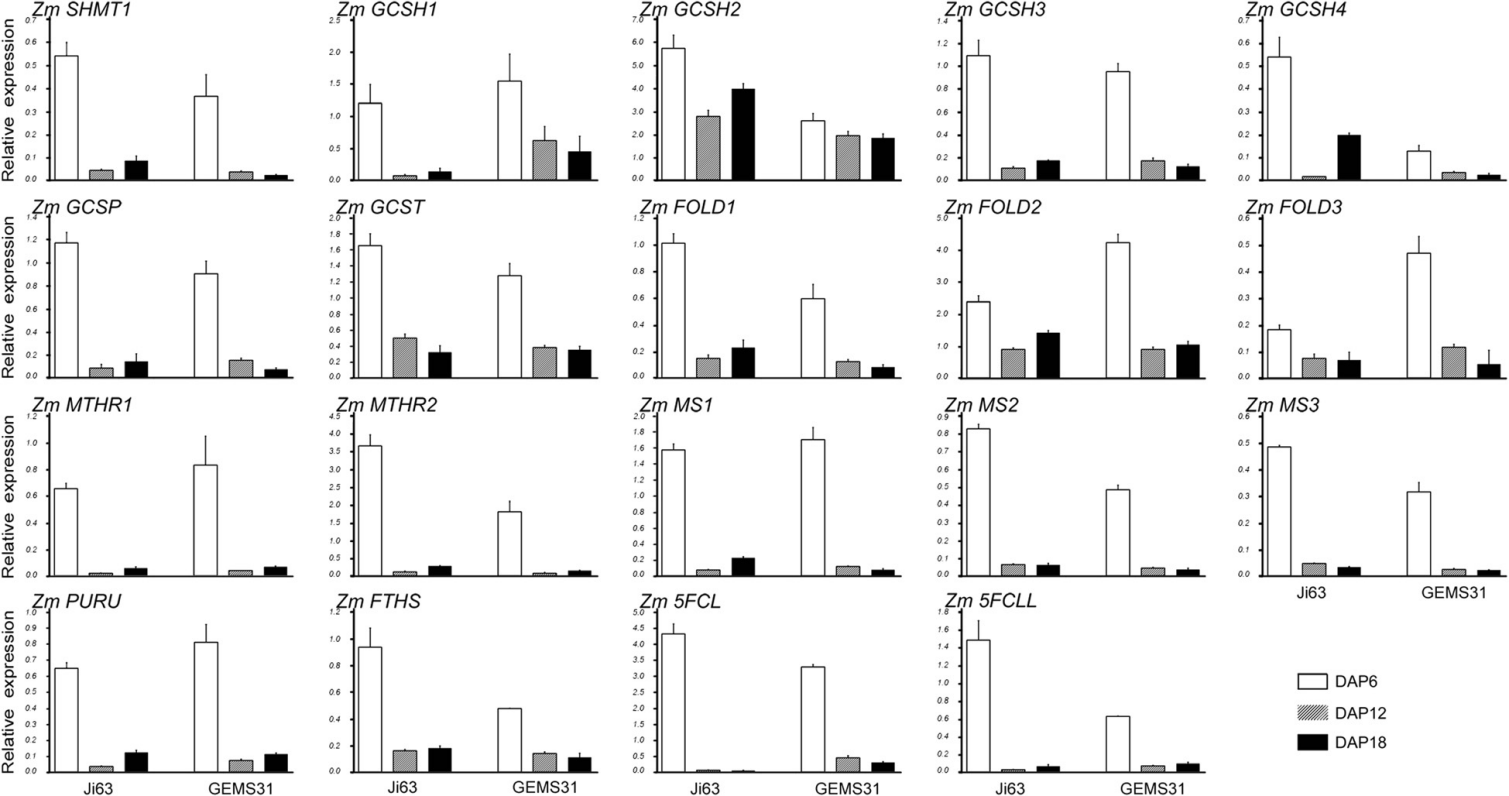
qRT-PCR of C1 metabolism related genes during kernel formation. qRT-PCR of C1 metabolism related genes during kernel formation Ji63 and GEMS31, respectively. Three biological samples were used for analysis and all reactions were performed in quadruplicate. Data are means ± SD (n = 4). Names of the proteins are listed in Table 2. The same samples were used as that used for folate profiling

To understand the transcriptional expression of the genes involved in folate and C1 metabolism, the ortholog genes identified above were investigated in the developing seeds of Ji63 and GEMS31 using qRT-PCR (Figs. 6 and 7). The same samples were used as that used for folate profiling. Transcripts of the genes involved in folate biosynthesis were most abundant on DAP 6 in the two lines (Fig. 6), and a similar pattern was observed for C1 metabolism-related genes (Fig. 7), albeit an exception was observed for *ADCL2* in Ji63 (Fig. 6). The most active DNA synthesis takes place at early stage of seed development (DAP 1 to DAP 6), for which the folate-dependent purine and pyrimidine synthesis is required [31, 32]. Thus, the observation that the highest transcript levels of folate-related genes were detected on DAP 6 is supportive of the previous reports, and indicates that the folate and C1 metabolism is active in young seeds.

However, a precaution must be taken to correlate the gene transcript levels with folate levels. First, the folate profiling revealed a peak of 5-M-THF on DAP 12, but transcripts of the genes encoding MS, consuming 5-M-THF to synthesize methionine, and MTHFR, catalyzing formation of 5-M-THF, peaked on DAP 6 and decreased sharply on DAP 12 and DAP18 (Figs. 5 and 7). Second, there was no significant difference in transcript abundance of the folate-related genes between GEMS31 and Ji 63 although the total folates in the dry seeds were markedly different. The observations mentioned above suggest an existing complicated folate metabolism-regulatory mechanism in maize seeds. Investigation of the enzymatic activities of folate-related enzymes in combination with a genome-wide association study would allow us to elucidate the roles of the folate metabolism-related proteins in folate derivative accumulation in maize kernels.

## Conclusions

Taken together, these findings suggest that folate and C1 metabolism is conserved between maize and other species, especially sorghum and millet. Metabolite profiling demonstrates that 5-M-THF is the dominant folate derivative in early developing seeds, and 5-F-THF is the major storage form in mature seeds. These two folate derivatives play different roles during kernel development. Genes involved in folate and C1 metabolism are actively expressed at the early stages of kernel development. This study provides a foundation for a future in-depth investigation of folate metabolism in maize.

## Methods

### Plant materials and folate measurement

Ji63 and GEMS31 inbred plants were grown at Shunyi, Beijing, China in the summer of 2013. The experimental field was loamy soil with pH 6.8, organic matter 0.7 %, phosphorus 13.8 mg/L, and potassium 48 mg/kg. During field preparation, 440 kg/acre of urea (46-0- 0) was applied. The herbicides were applied 5 d after planting. Plants were hand planted in 5-m-long rows with row and plant spacing of 25 cm, respectively. Kernel samples were harvested on 6, 12, 18, 24, and 30 days after pollination (DAP) and removed from the ear axis of three ears, respectively. Three biological replicates which the kernels from three ears were mixed as one replicate were harvested and frozen in liquid nitrogen immediately. The folates exaction and measurement were repeated for four times in each replicate. Similar results were obtained in these replicates, and the results of one replicate were described and discussed in this reports. Besides, these two inbred lines were grown in 2009 in Hainan, in 2010 in Yunnan, and in 2012 in Hainan, China.

Standards of 5-M-THF and 5-F-THF were purchased from Schircks Laboratories. The samples collected from field were used for identification of folate profiles. The methods for sample preparation and metabolite measurement were described previously [20]. The contents of folate in dry seeds of each inbred line were measured once across the four consecutive years. Folates in seeds on DAP 6, 12, 18, 24, and DAP 30 were measured in four biological replicates, and each sample consisted of 50 mg of plant material.

### Identification of folate metabolic genes in maize and other species

With reported processes of the folate metabolic enzymes in plants as queries [3], the Blast software were used to search the maize genome databases, including the Maize Genetics and Genomics Database [33], Arabidopsis Information Resource [34], National Center for Biotechnology Information [35] (NCBI), Phytozome [36], and the Swiss-Prot Protein Database [37] (Swiss-Prot). The proteins and their accession numbers used for alignment and phylogenetic tree construction are listed in Table 3.

### Alignment, phylogenetic analysis and domain detection

Total of 238 amino acid sequences of folate metabolic enzymes in maize and other species were aligned using the ClustalW tool [38]. The multiple alignments resulted in an unrooted distance tree using neighbour-joining algorithms of MEGA version 5. The reliability of the tree was examined using bootstrap analyses (1000 replicates). The conserved motifs were identified using Simple Modular Architecture Research Tool [15].

### Quantitative real-time qRT- PCR

Total RNA from maize kernels of DAP 6, DAP 12, and DAP 18 was extracted using a standard TRIzol RNA isolation protocol (Invitrogen) [39], respectively. To eliminate any residual genomic DNA, total RNA was treated with RNase-free DNase I (New England Biolabs) [40] and used to synthesise first-strand complementary DNA (cDNA) using the RevertAid First Strand cDNA Synthesis kit (Fermentas) [41]. Primers used in this paper are listed in Table 7. Primer premier 5.0 [42] was used to design the primers according to the CDS sequences of related genes.

**Table 7:** Primers used for qRT-PCR

Gene abbreviation	Forward primer sequences (5′-3′)	Reverse primer sequences (5′-3′)
*ACTIN*	GGGATTGCCGATCGTATGAG	GAGCCACCGATCCAGACACT
*GCH1-1*	GGAGGAAAGCGACTACATCGG	GAAACAGAGCACCTTGCACTATG
*GCH1-2*	GCAAAGCGACTGCATCCC	CACCCCGCACTATGTCCTTC
*FOLB1*	GCGGCCTTCAGTTCCACG	CCTTTGCAATGCTGTAGATATCGG
*FOLB2*	CGCCTGGATAGACCTCGC	GAGGCTTGCCAACCTTCACT
*HPPK/DHPS*	TCTCATACGCTCAACCATGCTC	GGAACAACATGTCTGGAAGCTCT
*ADCS*	CTTGTGAGTCAGATGATAGCCGAG	AATCTGTCTTCCGTGATGAGTAGC
*ADCL1*	GAGCTTGGCATAGGCGAAC	CTCCCATACCACCAGGGTG
*ADCL2*	GTCAGCACCAGGGACATCACAG	CCCACAGCAGATCAGACAGCG
*ADCL3*	n/a	n/a
*DHFS-1*	CTCCGACGACGGGTTTGAC	CTCATGATATTGGACAGGAATGCAG
*DHFS-2*	CGCAAGGCTACAATGTGGG	AGAGAGCAGTAAAAACCTCAAAATG
*DRTS-1*	GAGAAAGTGTTTGTTATAGGAGGCG	CTGAGAAGTCAACCGGAGGG
*DRTS-2*	GTGATAGAGAGCAACATTAGGCATT	CGACAACACCACGCCAAAATACC
*DRTS-3*	CATGTTCGAGCACTGGAGGAGC	CATCTCTATCTTCTGGTGGGGGTC
*DRTS-4*	CAGTGGCTCAACAAATGCAAAG	TCCAGTATAGTCAGCATGCATGTC
*FPGS-1*	GCAGTTGAAAGTGGTTCACGTTG	CCATCAAGCCGAAATCGCTC
*FPGS-2*	ACGTTACCACTCAATCGTACTG	GGGAAAACCACTTGCCAC
*SHMT1*	CGCAAGATACTACGGGGGAAATG	TGAGAAAGATGTCCACCGTGAGG
*GCSH1*	CTATCCGATCCAACCCTTTC	CCGTCGTCTTGACCCATT
*GCSH2*	CGCCTACCTCAGGATCTCCAC	GGTCAGTAATCCCCACGGTTG
*GCSH3*	CGAACAACCCTCGTCCACC	CCCATTCATGAGTGTCAGCATAT
*GCSH4*	AGCGGGAGAGAGAGGAGCG	CTGGTCGCCTTCACGCTCTC
*GCSP*	CTCGCTATGCCACAGTATGATC	TAACAGGTTGCCCAAGTCGTC
*GCST*	CGGATGCAGGGACAAGGAC	CTCAAATCTTCTTTCGTCAGCAAC
*FOLD1*	GTTGCCTGGAAACTGTTCAGAAG	CATTTAAGGGATGGAAACCATC
*FOLD2*	AACATCGTCGGGCTACCT	CTGGCTTGATCCAGTCACCT
*FOLD3*	CGACTCAGCAACCGTCTCAG	CTGAGAATCCTTCCTCGACCC
*MTHR1*	TCGAGTACTTCCCTCCCAAG	CCACACACACCATGTTCTGC
*MTHR2*	TACAAGGCGAGGGAGGTG	CAAGTAATACCAATTTGGCGG
*MS1*	TACAATCGGTTCGTTCCCAC	GATTTCCTCCTTGATGGCAGT
*MS2*	GACCACCGCCGTTCTACC	CGACCTTGCTGATTTCTTCC
*MS3*	GAGGGTCCGTCGTGAGTAC	CCATCCGTTGGCAGTGAAT
*PURU*	CGGGGCAACTAGCCATTTCG	GGTAGGACACGAAGCTCGCAATATG
*FTHS*	CTACGACCTCTACGGCAAGTAC	GACGGAGGCAAGTGACAAC
*5FCL*	TGTCAGCAGTTGCGAGAAG	GTTCCCAGTAGCATCCACAG
*5FCLL*	ACGGTTAGGGAAGGGAGAGG	TGTGGCTTTGGGATCGTAGTC

qRT-PCR was performed in a 7500 real-time PCR system using the SYBR premix Ex Taq (TaKaRa) [43]. The cDNAs were made from three samples and all reactions were performed in quadruplicate. PCR conditions were as follows: 95 °C for 30 s, 40 cycles of 95 °C for 5 s, 60 °C for 34 s. The *ACTIN* (GRMZM2G126010) was used as the reference gene to normalize the target gene expression, which was calculated using the relative quantization method (2^-ΔΔCT^).

### Availability of supporting data

The phylogenetic data has been deposited in TreeBase [44], and the accession URL is: http://purl.org/phylo/treebase/phylows/study/TB2:S17972.

## Abbreviations

ADC: 4-aminodeoxychorismate
ADCL: ADC lyase
ADCS: ADC synthase
ARATH: Arabidopsis
C1: One-carbon
DAP: Day after pollination
DHC: 5,10-methylene-THF dehydrogenase/5,10-methenyl-THF cyclohydrolase
DHFR: Dihydrofolate reductase
DHFS: Dihydrofolate synthetase
DHN: Dihydroneopterin
DHNA: Dihydroneopterin aldolase
DHPS: Dihydropteroate synthase
ECOLI: E.coli
FPGS: Folylpolyglutamate synthetase
FTHS: 10-formyltetrahydrofolate synthetase
GDC: Glycine decarboxylase
GDCH: Glycine decarboxylase H protein
GDCP: Glycine decarboxylase P protein
GDCT: Glycine decarboxylase T protein
Gly: Glycine
GTPCHI: GTP cyclohydrolase
HPPK: Hydroxymethyldihydropterin pyrophosphokinase
LC/MS: Liquid chromatography-tandem mass spectroscopy
MS: Methionine synthesis
MTHFR: Methylenetetrahydrofolate reductase
ORYSJ: Rice
p-ABA: Para-aminobenzoate
Ser: Serine
SETIT: Millet
SHMT: Serine hydroxymethyltransferase
SMART: Simple modular architecture research tool
SORBI: Sorghum
THF: Tetrahydrofolate
5-FCL: 5-formyl THF cycloligase
5-FCLL: 5-formyl THF cycloligase-like protein
5-F-THF: 5-formyl THF
5-M-THF: 5-methyl-THF
10-FDF: 10-formyl THF deformylase
